# Advancements in transfer printing techniques and their applications in photonic integrated circuits

**DOI:** 10.1038/s41377-025-02064-w

**Published:** 2025-12-05

**Authors:** Can Yu, Meng Zhang, Lei Liang, Li Qin, Yongyi Chen, Yuxin Lei, Yubing Wang, Yue Song, Cheng Qiu, Peng Jia, Dabing Li, Lijun Wang

**Affiliations:** 1 State Key Laboratory of Luminescence Science and Technology, Changchun Institute of Optics, Fine Mechanics and Physics, Chinese Academy of Sciences, Changchun, 130033 China; 2 Daheng College, University of Chinese Academy of Sciences, Beijing, 100049 China

**Keywords:** Semiconductor lasers, Photonic devices, Silicon photonics

## Abstract

Transfer printing is a powerful and versatile integration method that is attracting increasing attention as regards both scientific research and industrial manufacturing. The transfer printing technique utilizes the viscoelastic properties of a stamp to pick devices (ink) from a donor substrate and print them onto a target substrate, exploiting the competition between several interfacial adhesion forces. The overall yield can be improved through the introduction of external stimuli such as light, heat, solution, pressure, and magnetic fields during the transfer printing operation. This review summarizes different transfer printing methods based on their working principles and discusses their detailed applications in photonic integrated circuits, taking lasers, semiconductor optical amplifiers, photodetectors, and other optical electronic elements as examples. Hence, the feasibility and viability of transfer printing are illustrated. Additionally, future challenges and opportunities for innovative development are discussed.

## Introduction

Transfer printing (TP) is an exceptionally potent integration technology, and has gained significant attention in academia and industrial manufacturing for its high accuracy, high fidelity, and low cost. Soft stamps, which are usually made of polydimethylsiloxane (PDMS), have elastomeric properties that can be exploited to retrieve device coupons or material films from a growth substrate and print them onto a target substrate. Various TP methods have been designed to accommodate different application scenarios, including photonic/electronic-device^1–9^, sensing-array^10,11^, and solar-cell^12,13^ fabrication, along with biomedical applications^14–17^. Appropriate stamp-structure design or the introduction of an additional interlayer during the TP operation modulates the adhesion strength between the interfaces, enhancing the yield^18,19^. This is a promising technique for the deterministic assembly of microscale devices, facilitating the integration of Ⅲ–Ⅴ non-native devices onto photonic integrated circuits (PICs) in a massively parallel and cost-efficient manner.

**Fig. 1:**
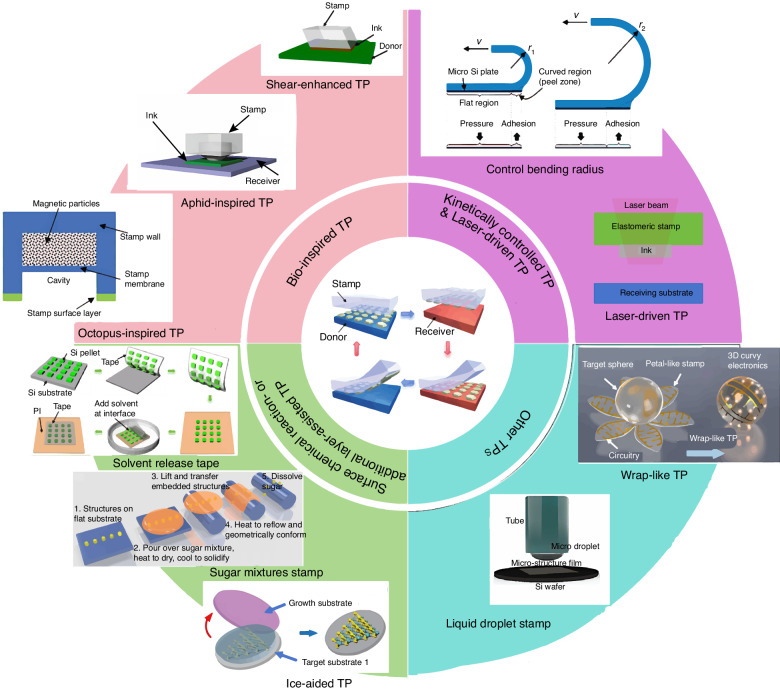
The summary of different TP methods discussed in this review. Reproduced with permission. Copyright 2005, Springer Nature^22^. Copyright 2016, American Chemistry Society^23^. Copyright 2015, Springer Nature^28^. Copyright 2022, the American Association for the Advancement of Science^33^. Copyright 2023, John Wiley and Sons^37^. Copyright 2012, John Wiley and Sons^56^. Copyright 2020, John Wiley and Sons^65^. Copyright 2021, John Wiley and Sons^69^. Copyright 2023, IOP Publishing; Institute of Physics Publishing Ltd^71^

This review aims to present a comprehensive overview of TP, highlighting the most recent advancements and offering guidance for future design of TP technologies with enhanced quality. Moreover, we also discuss their detailed applications in PICs. Figure 1 summarizes the TP methods discussed herein according to their intrinsic properties. This review is arranged as follows: Sections “Kinetically controlled TP”, “Surface chemical reaction- or additional layer-assisted TP”, “Laser-driven non-contact TP”, “Bio-inspired TP” and “Other TP techniques” introduce diverse TP methods, providing application-based selection guidance (Table 1). The section “Applications” considers practical applications of certain approaches, verifying the feasibility and reliability of TP. Finally, the future challenges for TP and associated opportunities are discussed.

**Table 1:** Summary and comparison of different TP methods

Approach	Stamp material	Regulated parameter	Adhesion switching ratio	Application	Ref.
Kinetically controlled TP	Elastomeric polymer	Peel-off velocity	~3	Au thin film Si/GaAs micro-structure	^20–22^
	Elastomeric polymer	Bending radius	/	Microscale Si plate	^23^
Surface chemical reaction- or additional layer-assisted TP	Elastomeric polymer	Amount of chemical bonds and glue-layer category	/	GaAs/InP micro/nano wire arrays	^24^
	Thermal release tape	Temperature	Infinite	Soft neutral electrode arrays	^25–27^
	Solvent release tape	Solvent category	>200	Si plate arrays Si photodetector arrays EMG sensor	^28^
	Photo-sensitive tape	UV-light intensity and exposure time	117.5	Au film GaN arrays micro-LED arrays	^29^
	PNIPAAm	Temperature	/	2D gold nanoparticle arrays	^30,31^
	Polyvinyl-alcohol stamp	van der Waals force	>10^6^	Cu Au Ag Pt Ti Ni	^32^
	Sugar mixture stamp	Amount of solvent and temperature	Infinite	Long and thin metal strips Au disks	^33,34^
	Thermal release tape with water serving as an adhesive layer	Category of certain metal or metal diodes	/	Nano-wire resistor	^35,36^
	Ice	Temperature	/	2D planar or continuous material	^37^
	Elastomeric polymer	Category of soluble interlayer or low surface-energy metal film	/	Micro liquid metal electrode Au nanowire arrays capacitor and LED circuit	^38–42^
Laser-driven non-contact TP	Elastomeric polymer	Laser intensity and exposure time	Infinite	Si chip Si platelet micro-LED	^43,44^
	SMP embedded with carbon black particles	UV exposure	Infinite	Si gold-coated Si	^45–47^
	Stamp with cavity filled with air and encapsulated by thin film	UV exposure	Infinite	Si platelet micro-LED arrays	^48^
Bio-inspired TP	Gecko-inspired stamp	Lateral or vertical shear force	~204	Si thin membrane Si platelet	^49–55^
	Aphid-inspired TP	Contact area	>1000	Si plate Si thin membrane	^56–61^
	Octopus-inspired stamp	Cavity pressure	~293	InGaAs nano-film/nano-ribbon Si micro-ribbon Si wafer PI thin film	^62–68^
Other TPs	Liquid drop stamp	Liquid volume	<25	Inorganic flexible thin film micro-LED	^69,73^
	Balloon stamp	External pressure	/	Si platelet Si-based solar cells Si-based photodetector	^70^
	Wrap-like stamp	External pressure	/	Light-emitting arrays solar cells	^71,72^

## Kinetically controlled TP

The adhesion strength can be modulated using a kinetically controlled TP technique. Viscoelastic elastomeric stamps can interact with ink via van der Waals forces to form an adhesion state. Mechanical manipulation has modulated the elastomer stamp/ink interfacial adhesion strength for ink pick-up and release; for example, the delamination speed and bending radius of the stamp have been adjusted.

**Fig. 2:**
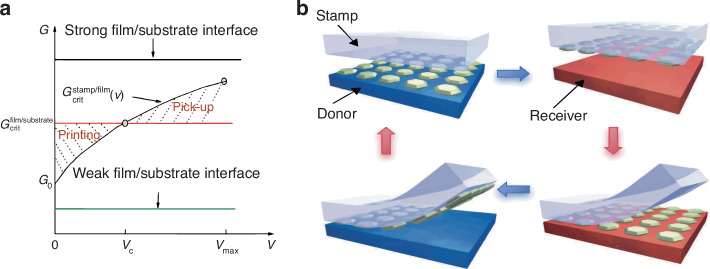
The basic mechanism of stamp delamination speed controlled TP method. **a** Schematic diagram of critical energy release rates for device/substrate and stamp/device interfaces^20^. Reproduced with permission. Copyright 2007, American Chemical Society. **b** Schematic illustration of TP process: retrieval at high velocity and printing at low velocity^22^. Reproduced with permission. Copyright 2005, Springer Nature

The TP process can be viewed as two competitive interfacial force fractures: the stamp/device and device/substrate forces. As the stamp is viscoelastic, the stamp/device interface critical energy release rate, ${G\,}_{\text{crit}}^{\text{stamp}/\text{device}}$, depends on the peel-off velocity. The substrate and device are elastic; therefore, the critical energy release rate for the device/substrate, ${G\,}_{\text{crit}}^{\text{device}/\text{substrate}}$, is independent of the peel-off velocity. In 2007, Feng et al.^20^ demonstrated that ${G\,}_{\text{crit}}^{\text{stamp}/\text{device}}$ increases monotonically with the separation velocity, enabling modulation of the interfacial adhesion force through velocity adjustment^20,21^. When ${G\,}_{\text{crit}}^{\text{stamp}/\text{device}}={G\,}_{\text{crit}}^{\text{device}/\text{substrate}}$, there is a critical separation speed corresponding to the intersection of the two curves (Fig. 2a). If the peel-off velocity exceeds the critical separation speed, pick-up occurs. If the critical separation speed exceeds the peel-off velocity, printing occurs. Accordingly, devices can be retrieved from the donor substrate at high peel-off velocities and printed onto a target substrate at low velocities, typically 10 cm s^−1^ and 1 mm s^−1^, respectively (Fig. 2b)^22^. This is a simple and convenient TP operation; however, the adhesion state switchability is limited, and device pick-up from strong device/substrate interfaces and printing onto weak device/substrate interfaces are difficult.

**Fig. 3:**
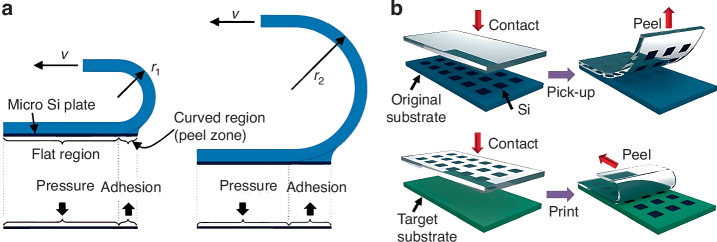
The basic mechanism of stamp bending radius controlled TP method. **a** Schematic diagram of adhesion state induced by elastomeric-stamp bending radius. Reproduced with permission. Copyright 2016, American Chemistry Society. **b** General TP process of Si-plate arrays onto glass glide^23^. Reproduced with permission. Copyright 2016, American Chemistry Society

Controlling the elastomeric-stamp bending radius also enables microscale device pick-up and printing. In 2016, Cho et al.^23^ developed a non-adhesive interlayer TP method to deliver Si plate arrays with sizes ranging from tens of micrometers to several millimeters onto various substrates. The interface adhesion strength is controlled by the stamp bending radius: a smaller (larger) bending radius induces a weak (strong) adhesion force, facilitating printing (device retrieval from the donor substrate) (Fig. 3a). Hence, Si plate arrays were successfully delivered onto glass plates (Fig. 3b)^23^.

TP controlled by kinetically switchable adhesion is simple and effective^20^. However, a sophisticated machine is required to control the peel-off velocity under the rate-dependent adhesion effect of the stamp, which increases the cost. Moreover, the limited adhesion switchability restricts the application range.

## Surface chemical reaction- or additional layer-assisted TP

A surface chemical reaction or additional layer can assist TP. The surface chemical reaction involves a bonding reaction and the intrinsic properties of the ink material, e.g., its hydrophobicity or hydrophilicity. The additional layer may comprise glue, various responsive tapes, graphene, N-isopropylacrylamide (PNIPAAm) coating, mixed sugar, a sacrificial layer, etc. For reliable, clean, and high-fidelity TP, the temperature change, chemical solution, bond density, or sacrificial layer should be carefully considered.

For reliable TP, the introduction of a chemical reaction and glue layer is feasible. For example, Sun and Rogers^24^ utilized condensation reaction and glue layer to deliver GaAs wire arrays onto a plastic substrate. To minimize the damage to the device performance, the chemical-bond density should be carefully designed.

**Fig. 4:**
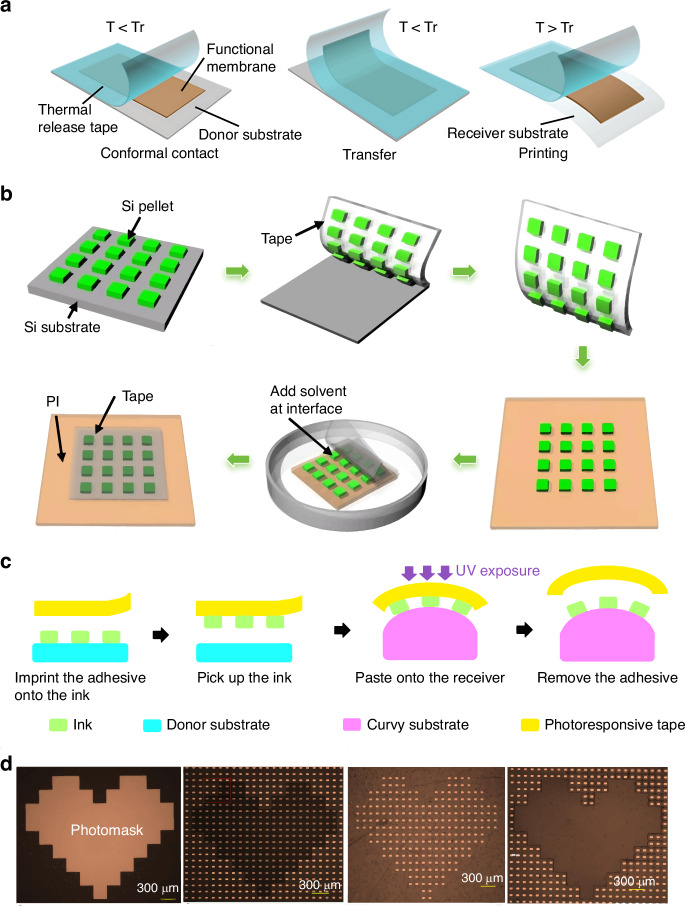
The basic mechanism of responsive tapes as stamps for TP operation. **a** The mechanism of thermal-release TP process^26^. Reproduced with permission. Copyright 2017, John Wiley and Sons. **b** Solvent release TP process^28^. Reproduced with permission. Copyright 2015, Springer Nature. **c** Schematic TP process on curved surface^29^. Reproduced with permission. Copyright 2022, Springer Nature. **d** Selective transfer process of GaN arrays onto PET substrate^29^. Reproduced with permission. Copyright 2022, Springer Nature

Thermal release tape (TRT) is thin and flexible, and exhibits a large, switchable and irreversible change in adhesion strength when heated to ~100 °C^25^. The stamp/ink adhesion state transforms from strong to weak when heated to the transition temperature, ensuring reliable retrieval and printing (Fig. 4a). Using TRT, in 2007, Yan et al.^26^ fabricated a stretchable conformal neural electrode array. In 2009, Ishikawa et al.^27^ utilized transfer-printed nanotubes as active channels to fabricate highly effective-mobility fully transparent thin-film transistors (~1300 cm^2 ^V^−1^ s^−1^)). Owing to its flexible strong-to-weak adhesion switchability, cost-effectiveness, and temperature-controllable properties, using TRT as printing stamp is considerably easier than conventional TP. However, this method is not suitable for thermally sensitive devices because heat application may degrade the device performance.

Solvent release tape (e.g., 3M3850 tape and acetone solution) is also used. Sim et al.^28^ reported that, for immersion of a receiver substrate with an inked stamp in acetone, the tape lost adhesion and simultaneously separated from a polyimide film (PI) substrate and silicon pellets, printing the silicon pellets on the PI substrate (Fig. 4b). Experiment revealed an overall transfer yield of 100% with no tape residue, indicating viable and high-fidelity TP. In some cases, the solution leaves residuals on the ink, potentially compromising device performance. Thus, both material properties and post-cleaning procedures must be considered for successful TP.

Photosensitive tapes display similar properties to TRT and solvent release tape. The photochemical crosslinking reaction induced by ultraviolet (UV) light weakens the adhesion state, enabling easy placement of the device on the receiver substrate (Fig. 4c). Hence, in 2022, Guo et al.^29^ successfully transferred wafer-scale gold membranes onto a PO substrate. To expand the application range, they also developed a photo-triggered selective printing method and realized selective GaN-array transfer (Fig. 4d). Moreover, this technique is compatible with curved surfaces. An ultra-thin Au membrane at 40 × 70 μm^2^ was delivered onto a curvy glass-bottle surface. Micro-LED arrays can also be transferred from silicon to glass substrates.

The three tapes mentioned above are responsive materials having strong and weak adhesion states at room temperature and under external stimuli (light, heat, a certain solution), respectively. These methods are cost-effective with minimal device damage; however, the irreversible change in tape adhesion strength is a major limitation.

**Fig. 5:**
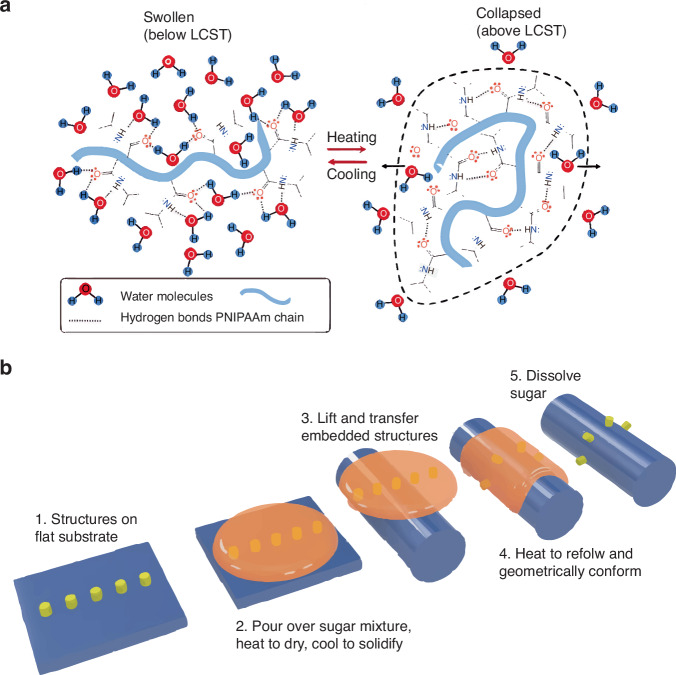
The basic mechanism of TP operation assisted by PNIPPAm and sugar mixtures. **a** Thermos-responsive behavior of PNIPPAm^30^. Reproduced with permission. Copyright 2023, John Wiley and Sons. **b** TP process of sugar mixtures as a stamp^33^. Reproduced with permission. Copyright 2022, the American Association for the Advancement of Science

PNIPAAm is a synthetic polymer that undergoes a phase transition from a hydrophilic to hydrophobic state when heated above its lower critical solution temperature (Fig. 5a)^30^. Its reversible changes in surface wettability have been used to make TP stamps. The pickup and release mechanisms were controlled by thermally sensitive changes in the PNIPAAm surface energy. The hydrophilic state (strong adhesion), enables pick-up, whereas the hydrophobic state (weak adhesion) facilitates printing. In 2017, the AuNP arrays had been transferred from a sacrificial Si wafer substrate to a receiver Si wafer with PNIPAAm coating successfully^31^. Utilizing the weak van der Waals forces of graphene, Liu et al.^32^ demonstrated a graphene-assisted method of transferring wafer-scale metal electrode arrays with a water-soluble polyvinyl alcohol stamp.

The TP approaches mentioned above are not suitable for casual 3D objects; thus, a novel method of 3D microprinting onto high-curvature surfaces is urgently required. In 2022, Zabow et al.^33^ presented a new TP method employing sugar mixtures as stamps that transform between solid and liquid phases on demand (Fig. 5b). The entire structure is heated to induce water evaporation. The inked sugar mixtures solidify after the device and sugar come into contact. The solution is then heated slightly, and the sugar stamp is dissolved in water to fully release the devices onto the target substrate. The authors demonstrated other inks, such as Au disks, printed onto various substrates, such as hair, leaves, red blood cells, poppy seeds, and floss fibers. The transparency of the ink-embedded sugar may also benefit alignment in the subsequent steps^34^.

**Fig. 6:**
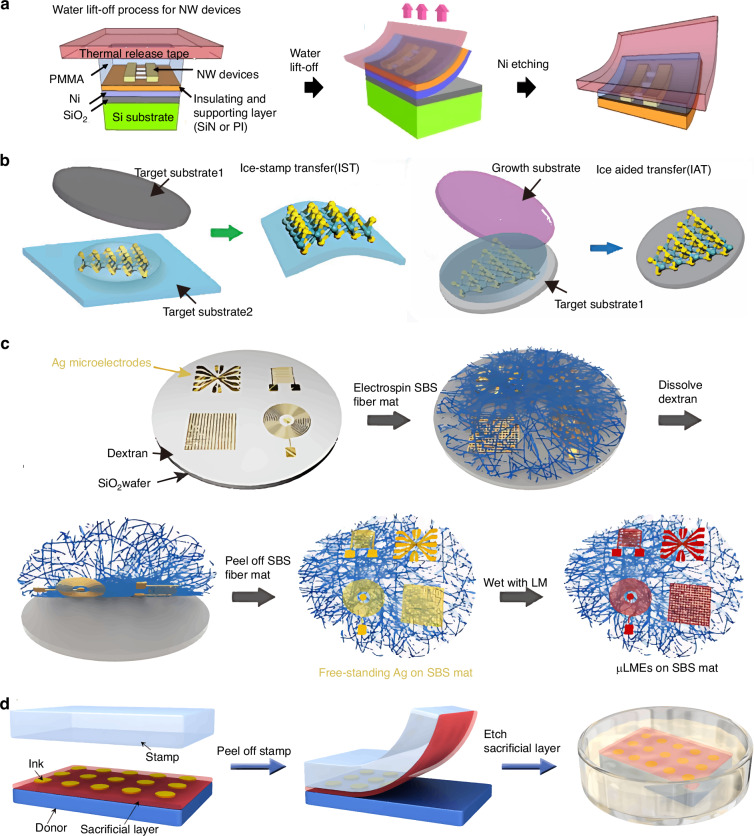
The basic mechanism of sacrificial layer assisted TP operation. **a** Water-assisted transfer procedures of nanowire devices^35^. Reproduced with permission. Copyright 2011, American Chemical Society. **b** IAT and IST^37^. Reproduced with permission. Copyright 2023, John Wiley and Sons. **c** TP process of Ag microelectrodes with dextran sacrificial layer^41^. Reproduced with permission. Copyright 2023, the American Association for the Advancement of Science. **d** TP process with Cu film serving as a sacrificial layer^42^. Reproduced with permission. Copyright 2020, Springer Nature

In 2015, Lee et al.^35^ proposed a wafer-scale water-assisted TP method that delivers nanodevices onto diverse and conventional substrates (e.g., paper, plastic, glass, and PDMS)^36^ at room temperature. The underlying principle of water-assisted TR is that water can react with certain metals or metal oxides (e.g., aluminum or nickel) to form soluble hydroxides. The general water-assisted transfer process for nanowire devices is illustrated in Fig. 6a. No residual material appears on the substrate or device, ensuring high fidelity. However, this method is unsuitable for water-sensitive materials or devices^34,35^.

Inspired by the above concept, in 2023, Liu et al. developed ice-aided transfer (IAT) and ice-stamp transfer (IST) to maintain cleanliness during TP (Fig. 6b); these techniques can be applied to various 2D flakes and continuous 2D films^37^. IAT/IST guarantees exceptional cleanliness and high quality because ice is the only process medium. The ice adhesion is modulated by adjusting the freezing temperature. However, ice has limited adhesion switchability, and the IST/IAT yield requires improvement.

For pick-up, use of a sacrificial layer is the prevalent method for separating the ink from the donor substrate. The two conventional sacrificial-layer types used for pick-up: are a soluble layer that directly separates interfaces and certain low-surface-energy metal films^34^. Using polymethyl methacrylate as the sacrificial layer is suitable for transferring graphene micro sheet onto silicon-on-insulator (SOI) waveguide to form waveguide-integrated graphene photodetector with micrometer-level precision, which was demonstrated by Wang et al.^38–40^. Owing to graphene’s ultrahigh mobility for both electrons and holes and its ability to uniformly absorb light ranging from ultraviolet to infrared, the photoresponsivity of the integrated photodetector exceeded 0.11 A W^−1^ and absorption coefficient was estimated to be 0.27 dB μm^−1^, enabling most optoelectronic applications. Figure 6c shows the general process of Ag microelectrode delivery from SiO_2_ substrate to a fiber mat^41^. Cu thin films are commonly used as interface layers between donor substrates and devices to facilitate separation, owing to their low surface energies. In 2020, Liu et al.^42^ demonstrated nano-scale-ink transfer to a receiver substrate with a Cu-film sacrificial layer (Fig. 6d). Adopting a Cu sacrificial thin film has facilitated transfer of various devices onto receiver targets, including Au nano-line arrays, capacitors, and LED circuits. This sacrificial film facilitates TP process and protects ink from bending or cracking during pick-up. However, dissolution of the sacrificial layer may damage the device unpredictably degrading its performance. Moreover, a suitable solution that does not react with or dissolve the substrate is essential.

## Laser-driven non-contact TP

Lasers are widely used as external TP drivers that facilitate infinite adhesion switchability^21,43^. Laser-driven non-contact TP exploits the thermal-mechanical mismatch at the ink/stamp interface upon heating by a laser pulse. The stamp and device thermal response behaviors vary; this triggers a distinct, gradual, and automatic detachment of the device from the stamp edge^34^.

**Fig. 7:**
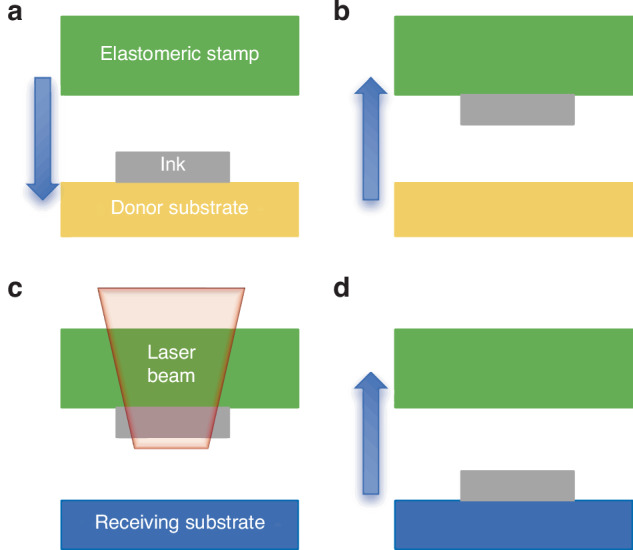
Laser-driven non-contact TP process. **a** Pick up ink from donor substrate with elastomeric stamp. **b** The separation between ink and donor substrate. **c** Ink release from stamp to target substrate assisted by laser pulse. **d** Lift stamp to print ink onto target substrate

The general process of non-contact laser-driven TP is shown in Fig. 7. A Si chip is prepared on a growth substrate and picked using an elastomeric PDMS stamp. After alignment, the inked PDMS stamp is positioned a few micromillimeters above the target substrate. A pulsed laser drives the ink release from the growth substrate to the target substrate. Again, the pick-up process exploits the strong van der Waals force between the stamp with posts and device. During printing, the stamp does not directly contact the receiver substrate; thus, the printing process is independent of the receiving-surface topography and properties; this significantly broadens the range of compatible materials^44^. However, high temperatures may adversely affect the device's performance.

**Fig. 8:**
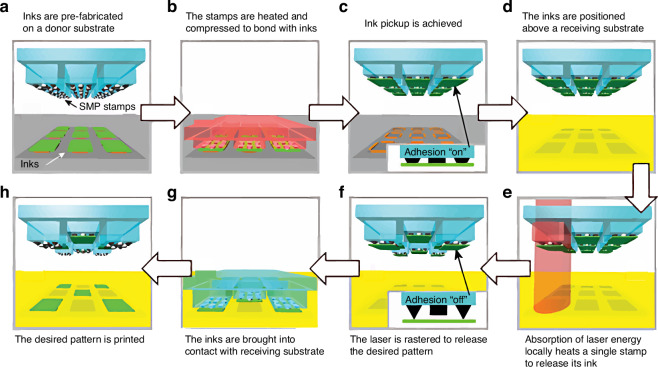
Laser-driven TP process using SMP stamp with carbon-black particles^45^. **a** Inks fabrication on donor substrate. **b** Heat and compress stamps to bond with inks. **c** Ink pickup. **d** Ink alignment. **e** Release ink from stamp with laser. **f** Raster laser to release desired pattern. **g** Contact inks with receiving substrate. **h** Print desired patterns onto receiving substrate. Reproduced with permission. Copyright 2016, John Wiley and Sons

In 2016, Eisenhaure and Kim^45^ proposed refined non-contact laser-driven TP that utilized shape memory polymers (SMP) and carbon black composite microstructures to reduce input laser power required for printing, thereby reducing the risk of device damage. SMPs can memorize temporary shapes and recover their original forms upon external stimulation (e.g., light or heat)^46,47^. Embedding carbon-black particles onto an SMP stamp makes heat localization and fast absorption feasible. Selective TP process of Si plate arrays onto a receiver target is shown in Fig. 8. Embedding carbon-black particles in the stamp shortens the heating time and reduces the required input laser power. However, elastomeric stamps require complex fabrication, an evident drawback.

**Fig. 9:**
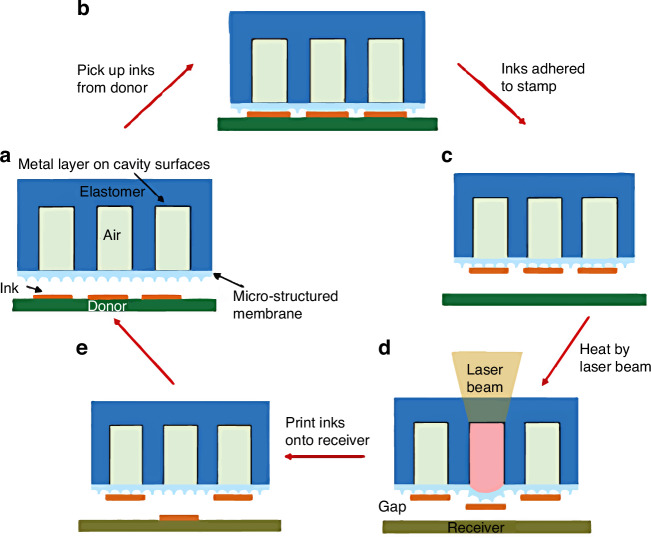
Laser-driven non-contact TP via micro-structured stamp embedded with cavity^48^. **a** Inks fabrication on donor substrate. **b** Pick up inks from donor. **c** Inks are adhered to stamp. **d** Heat by laser beam. **e** Print inks onto receiver. Reproduced with permission. Copyright 2019, Oxford University Press

In 2020, Luo et al.^48^ proposed a novel laser-pulse-driven TP method, where the stamp incorporates an air-filled cavity encapsulated by a patterned thin membrane (Fig. 9). The device/stamp interface adhesion state is modulated by the cavity pressure change. Locally heat-inked stamps introduce regional shape changes in the patterned membrane, enabling easier delamination between the stamp and device. For programmable TP operation, a local laser beam delivers the required patterns onto the receiver substrate. However, cavity volume adjustment can induce inaccurate displacement and reduce the overall precision^29^.

In summary, laser-driven TP renders non-contact operation feasible; this has not been achieved by other techniques^18,21^. Thus, the printing success is independent of the target-surface topography and properties, and a wider range of materials are compatible. Additionally, local laser-beam heating enables programmable TP; the required patterns can be delivered onto the receiver substrate with high resolution and accuracy^44,45,48^. Despite these merits, several issues remain. First, during TP, a sophisticated and costly machine is required to control the lasing-pulse intensity and exposure time, rendering this TP complex and expensive; this limits the development of industrial applications. Moreover, the parameters of each material vary; therefore, certain parameters should be tailored to specific conditions^34^. Moreover, this method is unsuitable for thermally sensitive devices or materials, because the high temperatures required may influence the overall performance.

## Bio-inspired TP

Certain creatures exhibit remarkable adhesion abilities tunable to the environment. The setae of gecko toes, pulvilli of aphid legs, and suckers of octopus wrists enable strong switchable adhesion and have inspired artificial adhesive research yielding elastomeric stamps with controllable surfaces. This section focuses on three bio-inspired TP techniques: gecko-^49^^–^^55^, aphid-^56^^–^^61^, and octopus-inspired TP^62–66^. Gecko-inspired TP relies on shear-force-induced directional adhesion. Aphid-inspired structures control adhesion through contact-area variation. Octopus-inspired TP simulates sucker states via external force-induced pressure differences.

### Gecko-inspired TP

The gecko has evolved one of the most effective and versatile known adhesives. The millions of setae arrays on its toes provide strong van der Waals forces to allow climbing on various surfaces^51^. The adhesion state between interfaces is direction-dependent, depending on the directional angle, which varies with movement of the gecko’s toe^49^. Gecko-inspired TP structures have been proposed, including the angled micro-flap stamp^50^, shear-enhanced TP^52^, stamps with posts^52,53^ or angled micropillars^54^, and stamps covered with natural setae arrays^55^.

**Fig. 10:**
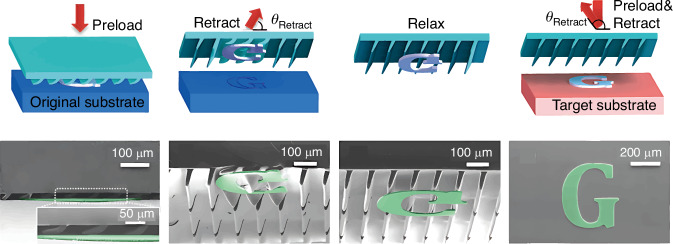
TP of Si membranes onto target substrate with angled micro-flap stamp^50^. Reproduced with permission. Copyright 2014, American Chemical Society

In 2014, Yoo et al.^50^ proposed an elastomeric angled micro-flap stamp for massively parallel delivery of multiple Si membranes of different sizes onto a target substrate. The stamp with compliant slanted micro-flap arrays possesses strong retraction-angle-dependent adhesion strength^50,67^ (Fig. 10). For maximum adhesion, the angled micro-flap stamp collapses with the preload and makes side contact with the Si membrane. The elastomer stamp recovers its original shape because of its viscoelastic properties; its tip attaches to the Si membrane. The inked stamp is pressurized onto the receiver substrate. This method enables simultaneous TP of multiple Si membranes of micrometers to millimeters. Further, no adhesive interlayer is required on the receiver substrate, reducing the risk of device contamination.

In shear-enhanced TP, a shear strain is applied to an elastomeric stamp as an alternative to adhesion-strength modulation. This approach was proposed by Carlson et al.^52^ in 2011, for an elastomeric stamp with a single, rectangular post. Pick-up is similar to that for kinetically controlled TP: high peel-off velocity separates the ink from the donor substrate. After target-substrate alignment, the inked stamp and receiver target make contact. Shear strain is applied to the stamp through lateral motion of the target substrate, reducing the normal component of the delamination force and facilitating efficient ink release from the stamp to the receiver target.

Based on vertical-post stamps, in 2012, Yang et al.^53^ presented a refined angled-post stamp with improved yield. The adhesion state was regulated by the retraction direction, and the crack propagation was faster when the opposite retraction force was applied to the post inclination for easier printing. The elastomeric stamp with angled elastomer micropillars with flat or round tip endings is also gecko-inspired^54^. Vertical or shear displacement control places the tip in contact with the ink, greatly reducing the adhesion strength and facilitating ink release from the stamp to the target substrate. In 2014, Jeong et al.^55^ presented a stamp covered with natural gecko setae arrays. Retraction along the proximal direction enables retrieval because strong adhesion strength is required. Retraction along the distal direction enables printing. However, utilizing natural setae arrays is cruel and costly, greatly restricting the application scope.

This gecko-inspired TP technique exhibits excellent adhesion switchability through application of an external shear force to modulate the interface adhesion state on demand. The main disadvantage is the complicated fabrication process for microscale posts or pillars, which increases the cost. Additionally, lateral or vertical shear force-induced ink displacement decreases the TP resolution^21^.

### Aphid-inspired TP

Another widely used adhesion control strategy mimics the unique biological structure of aphids. Pulvilli and tibial muscles regulate adhesion through contact-area adjustment. On smooth surfaces, increased blood pressure enlarges the pulvilli, enhancing the contact area and adhesion strength for lifting. Tibial muscle retraction reduces contact and adhesion for easy release. Several related TP techniques exist, including inflatable stamps^56^, PDMS/SMP stamps with pyramidal micro-structures^57,58^, stamps with embedded magnetic-sensitive particles^59^ or expandable microspheres^60^, and SMP stamps embedded with stiff spheres^61^.

**Fig. 11:**
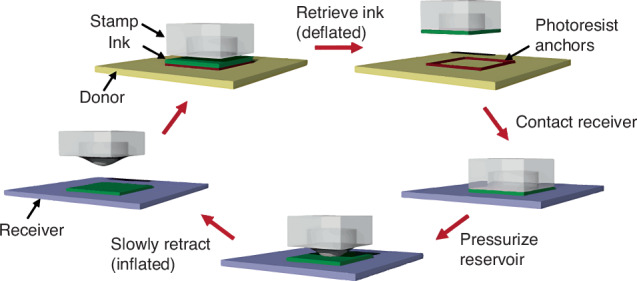
TP process with inflatable-stamp^56^. Reproduced with permission. Copyright 2012, John Wiley and Sons

In 2012, Carlson et al.^56^ proposed an inflatable stamp comprising an open reservoir and microchannels encapsulated by adhesive membranes. The adhesion strength is modulated by rate-dependent adhesion and actuation of subsurface fluid chambers (Fig. 11). This inflatable stamp has printed microscale Si plates onto various substrates (e.g., plastic sheets, leaves, and glossy business cards). Higher-reliability selective TP can also be realized through local pressurization of reservoirs to induce device separation from the stamp.

The stamp-surface pattern can also achieve controllable adhesion strength. In 2010, Kim et al.^57^ proposed surface-relief assisted TP for the deterministic assembly of microscale devices. Four pyramidal reliefs were grown on PDMS-stamp posts; the adhesion state was regulated by adjusting the contact area between the microtip and device surface. This technique can achieve high levels of control with a difference of more than three orders of magnitude between the strong and weak adhesion-state forces^25,57^.

A pyramidal-microstructure SMP stamp overcoming the PDMS-stamp limitations was proposed in 2014^58^. By utilizing the SMP shape-fixing and recovery properties through heating or cooling across the glass transition temperature, the adhesion strength is regulated for TP, with increased yield and no time sensitivity. The rigidity and shape are controlled by the application and absence of heat input. The maximum adhesion may increase dramatically for pick-ups performed at temperatures below the glass transition temperature, when the polymer is rigid.

In 2019, Linghu et al.^59^ presented an aphid-inspired design for elastomeric surfaces, including magnetically sensitive materials, having rapidly tunable and highly reversible adhesion strength. The stamp comprises a magnetic particle-filled cavity encapsulated by a patterned membrane; the adhesion state is regulated by contact-area changes under an external magnetic field. Five silicon platelets were successfully transferred from a donor substrate to a PDMS substrate.

**Fig. 12:**
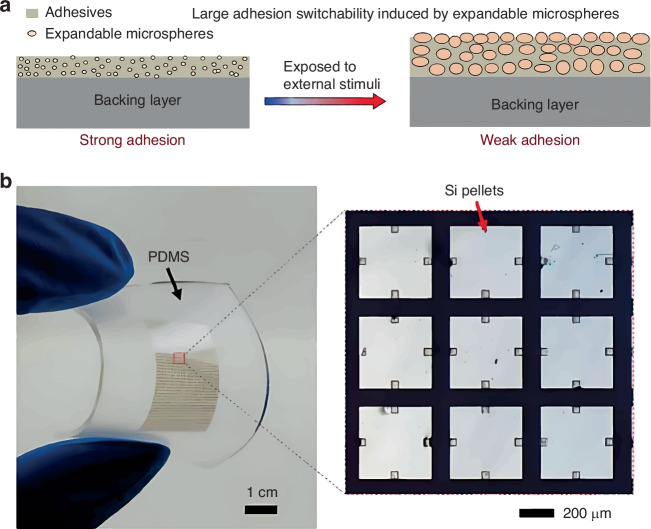
The basic mechanism of TP operation with shape-conformal stamp and the schematic of Si pellets on PDMS stamp. **a** TP process with expandable-microsphere-embedded stamp^60^. Reproduced with permission. Copyright 2020, the American Association for the Advancement of Science. **b** Thin Si pellet (400 μm × 400 μm × 200 nm) arrays on PDMS stamp^60^. Reproduced with permission. Copyright 2020, the American Association for the Advancement of Science

In 2020, Wang et al.^60^ developed a shape-conformal stamp with a polymeric backing layer and expandable microspheres embedded in an adhesive layer (Fig. 12a). The device/stamp-interface contact area is altered by external stimuli (e.g., light or heat) to adjust the adhesion strength. Ultrathin Si pellets (400 μm × 400 μm × 200 nm) were transferred from a growth substrate to a PI substrate (Fig. 12b). Local exposure to heat or laser beams enables this selective TP operation.

In 2021, Zhang et al.^61^ proposed an alternative design for thermally actuated switchable dry adhesives. The stamp comprises an SMP substrate with embedded stiff spheres, all encapsulated by an elastomeric membrane. The switchable adhesion state depends on rate-dependent kinetic control adhesion and the SMP shape fixing, and recovery properties^61^.

The core adhesion strength adjustment principle of the several aphid-inspired TP techniques is contact-area adjustment, which simulates the congestive or relaxed state of the aphid pulvilli. Although effective, these techniques require further improvement, particularly in terms of retrieval. If the device/substrate adhesion exceeds the stamp/device adhesion, pick-up fails, affecting the yield. Furthermore, microchannels and surface reliefs require complicated fabrication technologies and high accuracy. Finally, SMP or laser-beams based techniques require high temperatures; therefore, they are unsuitable for temperature-sensitive devices.

### Octopus-inspired TP

An octopus utilizes muscle actuation to adjust the internal or external sucker pressure. Hence, a strong or weak adhesion state is generated to attach to or release a foreign surface, respectively^62,63^. Various related TP techniques have been developed, including cavity-embedded stamps encapsulated by a PNIPAAm hydrogel layer^64^, stamps implanted with two air- and magnetic-particle-filled cavities separated by an elastic membrane^65^. The micro-structured stamp is also discussed here^66^.

**Fig. 13:**
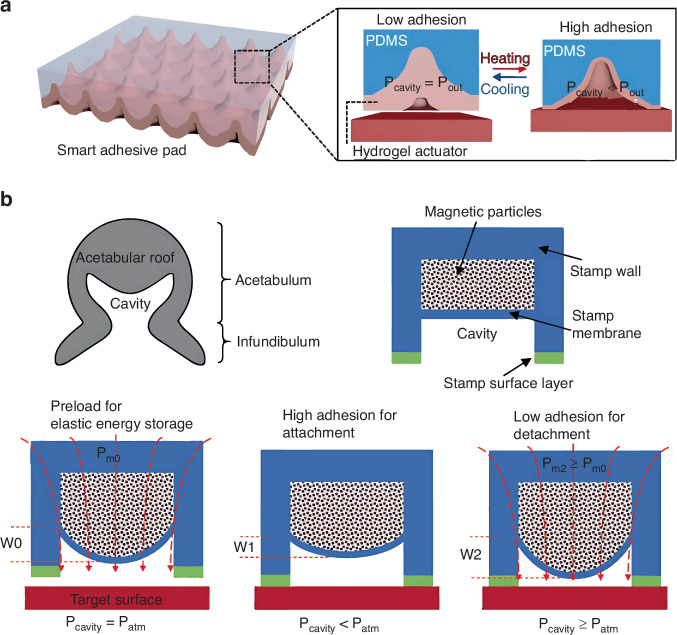
The basic mechanism of octopus-inspired TP. **a** Geometry of smart adhesive pad and corresponding TP mechanism^64^. Reproduced with permission. Copyright 2016, John Wiley and Sons. **b** Elastic energy storage enabled by magnetically actuated octopus-inspired smart pad^65^. Reproduced with permission. Copyright 2020, John Wiley and Sons

In 2016, Lee et al.^64^ presented a smart adhesive pad that actively controls adhesive strength via thermo-responsive actuation of a muscle-like hydrogel layer within a cavity. The PDMS stamp comprises an empty cavity encapsulated by a PNIPAAm hydrogel layer for thermos-responsive actuation (Fig. 13a). The PNIPAAm undergoes a phase transition from soluble to insoluble at a critical temperature (~32 °C)^68^. Below the temperature, the PNIPAAm-layer expansion reduces the cavity volume, increasing the cavity pressure and simulating sucker relaxation. Above the critical temperature, the PNIPAAm hydrophobicity increases the cavity volume, forming a pressure difference and strong adhesion for device lifting from the donor substrate. The smart pad exhibits an excellent on/off adhesive strength ratio (~293).

Although the smart adhesive pad has an excellent adhesion switch ratio, the device pick-up time from the donor substrate exceeds an hour. To overcome this, in 2020, Wang et al.^65^ demonstrated elastic energy storage enabled by magnetically actuated octopus-inspired smart pads. The structure features two elastic-membrane-separated cavities. The upper cavity is filled with magnetic particles, providing magnetic actuation under an external magnetic field; the lower cavity is empty, to control the pressure change between cavity interior and exterior (Fig. 13b). The strong/weak adhesion-state conversion occurs in milliseconds, significantly shortening the operative time. The pad is highly reversible, rapidly tunable, and switchable in both air and water. Another novel bionic-theory-based micro-structured stamp was proposed by Liang et al.^66^ in 2021, comprising a backing layer, square pillar, a square microchamber, and four microchannels. The core principle involves the pressure difference between the interior and exterior of the cavity induced by preloading. However, micro-structured stamps need complex fabrication technologies and nano-scale device transfer is still challenging.

Various structured stamps mimicking octopus’ suckers have been developed, where the pressure difference between the cavity interior and exterior are adjusted to convert the adhesion state on demand. However, these stamp designs require intricate fabrication procedures and an external actuation source is needed to generate a pressure difference, introducing additional expense and diminishing the overall accuracy.

## Other TP techniques

In addition to the conventional TP techniques above, novel methods aiming to improve overall TP yields and applicability have been developed, including liquid-droplet stamps^69^, balloon stamps^70^, wrap-like stamps^71^, and thermally triggered epoxy SMP blocks^72^. The ability to transfer three-dimensional devices onto curved substrates significantly broadens the application scope.

**Fig. 14:**
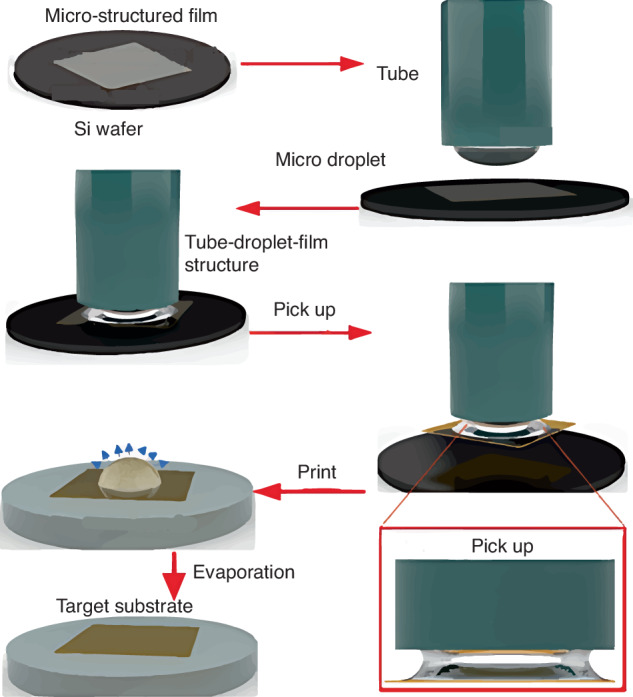
TP process of semiconductor film onto target substrate using liquid-droplet stamp^69^. Reproduced with permission. Copyright 2021, John Wiley and Sons

To reduce the device cracking or breaking risk caused by solid-contact elastomeric stamps, Liu et al.^69^ used a simple liquid-droplet stamp, in which a liquid bridge forms at the film/tube interface, to transfer a flexible thinned LED onto a PDMS substrate (Fig. 14). A hydrophilic rubber capillary tube functions as a handle to control the liquid-droplet volume^69,73^. Liquid bridges occur when the tube and film remain in intimate contact and pull the handle upward to retrieve the film from the growth substrate. Increase the droplet volume to break the liquid bridge for reliable printing operation. The film is printed onto the target substrate via van der Waals forces. Residual droplets are then evaporated from the film. High positioning accuracy is maintained without device damage; therefore, this technique may have broader applicability than conventional methods.

A 3D conformal curved stamp is required for patterning on 3D curved structures. In 2019, Sim et al.^70^ proposed a conformal additive stamp employing a pneumatically inflated elastomeric balloon as a conformal stamping medium to pick-up and print prefabricated electronic devices onto curved surfaces. Si photodetector arrays and Si-based solar cells can be printed onto hemispherical shells and PDMS substrates, respectively, with no damage to the device.

**Fig. 15:**
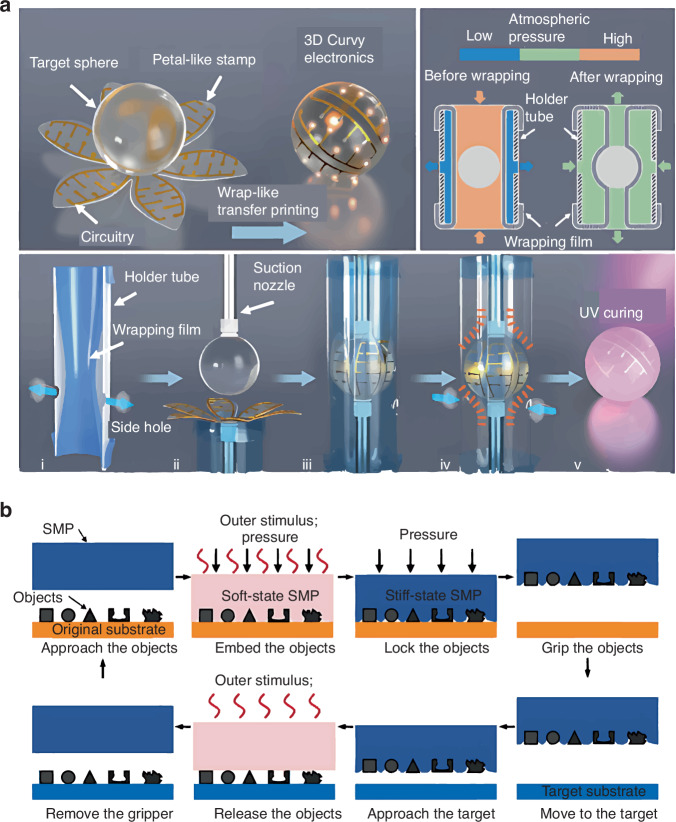
TP process with wrap-like stamp and SMP block. **a** TP process with wrap-like stamp^71^. Reproduced with permission. Copyright 2023, IOP Publishing; Institute of Physics Publishing Ltd. **b** TP process with epoxy SMP block^72^. Reproduced with permission. Copyright 2020, the American Association for the Advancement of Science

Another novel method for 3D curved electronics is wrap-like TP, demonstrated by Chen et al.^71^ in 2023. The petal-like stamp incorporates elastomeric rubber and water-soluble adhesive tape (Fig. 15a). Prefabricated planar circuits on the petal-like stamps can be printed onto a target sphere through uniform pressure from the strain recovery of a pre-strained elastic film. The approximate vacuum state in the wrapping film forces the petal-like stamp to tightly wrap the target sphere. Finally, UV illumination cures the target-sphere adhesive layer. The petal number and diameter can be refined through finite element analysis. The validity of this petal-like stamp was proven by fabricating spherical antennas, solar cells, and light-emitting diode arrays.

An SMP block can transfer 3D devices utilizing thermally triggered shape fixing; the SMP recovery property can handle objects ranging from tens of millimeters to tens of micrometers. Devices embedding into an SMP enables retrieval and shape recovery upon application of external stimuli, facilitating printing. Figure 15b illustrates the TP process using an epoxy SMP block. Micro-LED arrays have been transferred onto a PDMS substrate in a programmable manner using a local laser beam^72^.

## Applications

Silicon photonics (SiPh) is an established platform for realizing complex and powerful PICs^74,75^. Leveraging the high material quality and compatibility of SiPh platforms with mature complementary metal oxide semiconductor technology, SiPh has been applied to sensing^76^, signal processing^77^, quantum science^78^, telecommunications^79–81^, etc. However, native integration of functional optical elements and light sources (e.g., lasers, modulators, optical switches, tunable filters, photoelectronic detectors, and semiconductor optical amplifiers (SOAs)) has not been achieved. Therefore, many optical functions cannot be performed, which restricts the application range. Thus, III–V semiconductor devices must be integrated into PICs. Heterogeneous integration with multiple devices, enabling the desired functionalities on SiPh wafers, has been attempted. Epitaxial growth is promising, leveraging the best properties of III–V devices and advanced Si fabrication processes. Moreover, it enables precise control over thin film parameters, ensuring high crystal quality. However, the device reliability and performance require further demonstration and improvement^82,83^. Heterogeneous integration through wafer–wafer or die–wafer bonding allows low-loss evanescent optical coupling from III–V devices to SiPh circuits. This approach enhances system integration, making it suitable for large-scale mass production while reducing the packaging cost. However, the SiPh back-end flows must be modified and significant capital investment is required^84,85^. Flip-chip hybrid integration, where finished Ⅲ–Ⅴ device chips are directly assembled on SiPh enables independent Ⅲ–Ⅴ and Si optimization and qualification. However, the high packaging cost and limited alignment tolerance render this approach unsuitable for mass manufacturing and dense integration^85,86^.

This TP technique combines the merits of wafer bonding and flip-chip integration, enabling high throughput and device-quality pre-testing on the growth substrate, respectively. Wafer-scale integration of device coupons and material films is facilitated with minimal SiPh process-flow disruption. A comparison of the cons and pros of these integration methods are summarized in Table 2. Various Ⅲ–Ⅴ semiconductor devices have been transferred from donor substrates to PICs via TP without performance loss, including SOAs, lasers, detectors, modulators, tunable filters, and optical switches, broadening the PIC application range. Furthermore, several novel III–V devices have been designed based on this high-accuracy and low-cost technique. The following section discusses TP applications for SOAs, lasers, photodetectors, and other optical-electronic components integration on PICs.

**Table 2:** Comparison of different integration methods

Technology	Thermal budget	Alignment accuracy	Efficiency of III–V materials	Cost	Scalability	Throughput
Wafer bonding	High	High	Medium	Medium	High	High
TP	Low	Medium	High	Low	High	High
Flip-chip	Medium	Medium	Medium	High	Medium	Low
Epitaxial growth	Very high	High	Very high	Medium	Low	High

The most widely used TP method combines the elastomeric-stamp rate-dependent adhesion effect and gecko-inspired shear-enhanced TP. TP methods assisted by surface chemical reaction or additional layer may leave chemical residues, reducing the overall printing yield and degrading the device performance. For laser-beam- or magnetic-field-assisted TP, external machines monitor crucial parameters, increasing the complexity and cost. Bio-inspired stamps typically have complicated fabrication procedures and are still in early developmental stages.

X-celeprint μTP-100 is a mature lab-scale printer, with acceptable device performance, overall printing yield, compatibility with current technology, and cost^87^. The basic procedures for transferring Ⅲ–Ⅴ devices onto PIC with μTP-100 are described as follows. Typically, a recess in the back-end stack is necessary for a non-native device to reach the Si device layer or substrate on an SiPh platform. The spray-coated adhesive bonding layer (typically divinyl-siloxane-bis-benzo-cyclobutene) enhances the bonding strength between III–V devices and Si-waveguide. To break the tethers after device contact, the stamp is rapidly raised, detaching the device coupon from the growth substrate. The inked stamp is then placed in the PIC recess under slight pressure. For reliable printing of the device coupon on the PIC, the stamp is retracted slowly with shear force. Post-processing involves removal of the encapsulation and final metallization^87,88^.

### SOAs

SOAs are important for numerous PICs, and can act as post-transmission booster amplifiers and pre-amplifiers before receivers^89^. Their nonlinear effects, such as cross-gain and -phase modulation, can realize wavelength conversion^90^. Thus, the integration of high-gain or high-output saturation-power SOAs into PICs broadens their applicability. Examples are given below.

Haq et al.^91^ demonstrated a simple adiabatically tapered C-band SOA to handle state-of-art TP-tool misalignment. The Ⅲ–Ⅴ waveguide tapers from 3.2 to 0.5 µm over 225 µm; the Si waveguide beneath the Ⅲ–Ⅴ waveguide has 3-µm width. The Si waveguides are defined in a 400-nm silicon layer with a 180-nm partial etch. The III-V layer structure comprises a 260-nm-thick n-InP contact layer, an InAlGaAs active region with 6 quantum wells, and a 2-µm/285-nm -thick p-InP/p-InGaAs cladding. A 17-dB small signal gain was obtained at a 170-mA bias current at 1550 nm, indicating the feasibility of InP-SOA transfer onto the Si substrate.

**Fig. 16:**
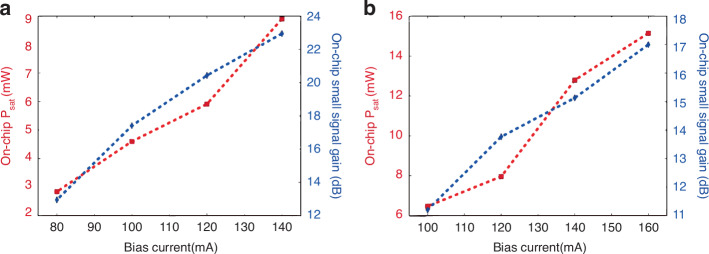
The performance of TP-SOA. Bias current as a function of on-chip saturation power (left) and small signal gain (right) for **a** full and **b** partial coupling designs at 1565 and 1548 nm, respectively^92^. Reproduced with permission. Copyright 2020, John Wiley and Sons

To tune small signal gain and output saturation power of SOA, in 2020, Haq et al.^92^ also reported micro-TP of a pre-fabricated C-band InP-SOA (1.35 mm × 40 μm) onto a PIC using an X-Celeprint μTP-100. Two different optical confinement factors in a quantum well enable tuning of a small signal gain and output saturation power for an SOA. An alignment-tolerant taper structure tolerates 1.0–1.5-μm lateral misalignment; a typical alignment accuracy when printing large-scale Ⅲ–Ⅴ device arrays. The designs with higher and lower confinement factors had 23- and 17-dB small signal gains, respectively, and 9.2- and 15-mW on-chip saturation powers, respectively, at 140- and 160-mA bias currents, respectively (Fig. 16a, b, respectively)^92^. A tradeoff exists between small signal gain and the output saturation power, consistent with theory.

In 2023, Soltanian et al.^93^ reported micro-TP-based Ⅲ–Ⅴ-on-Si SOAs with 15-dBm output saturation power having four segments: a taper for evanescent coupling from the single-mode Si waveguide to Ⅲ–Ⅴ, narrow side for high gain, wide side for high output saturation power, and inverted taper for evanescent coupling from the Ⅲ–Ⅴ to single-mode Si waveguide. At 1573 nm (the maximum optical gain) and 20 °C, the small signal gain was 9.4 dB and the output saturation power was 15.4 dBm.

Silicon nitride (SiN) waveguide platforms have wide optical transparency windows, rendering them suitable for PICs. A heterogeneously integrated InP/InAlGaAs-based SOA on a SiN platform with 14-dB gain and 8-mW saturation power was demonstrated in 2020^94^. A two-stage taper was designed for efficient light coupling from the SiN waveguide to the III–V stack layer via an interlayer a-Si:H layer. At 1570 nm, the SOA had 14-dB gain and 8-mW saturation power under 120-mA bias. Combining a 1-cm-long waveguide in the SiN layer with the amplifier output formed a laser cavity operating at ~1550 nm, demonstrating the amplifier potential.

Combining SOA with other optical devices can yield powerful functionalities. TP of two SOAs to a Mach-Zehner interferometer (MZI) circuit can achieve wavelength conversion, exploiting the SOA cross-grain and -phase modulation properties. Compared to a single SOA, this SOA-MZI exhibits a larger mainlobe-to-sidelobe ratio^95^. Cointegrating a Si-MZI switch and InP-SOA using TP enables a C-band lossless and high-speed Si-MZI switch^96^.

### Lasers

Integrated III–V lasers on SiPh platforms have evolved rapidly by leveraging low-loss passive Si waveguide and efficient TP techniques. The first published demonstrations of Ⅲ–Ⅴ lasers on silicon based on TP technique were achieved by Justice et al.^97^ and Yang et al.^98^ in 2012^99^. They realized a low-threshold current Fabry-Perot laser at 840 nm operating at temperatures up to 100 °C and an ultrathin membrane reflector vertical cavity surface-emitting laser (VCSEL) with power efficiency of <0.1% via two-step TP processes, respectively. Getting inspiration from this, various configurations have realized, high side-mode suppression ratios (SMSRs), high output powers, narrow linewidths, etc. A distributed feedback laser (DFB)^100^, DFB and optical power amplifier (OPA) co-integration^101^, a narrow-linewidth laser^87^, Fabry-Perot laser^102^, InAs/GaAs quantum dot laser^103^, and GaAs VCSEL have been used^104^.

In 2018, Zhang et al.^100^ demonstrated a Ⅲ–Ⅴ-on-Si DFB laser using TP. The DFB-laser epitaxial layer stack comprised a 200-nm p-InGaAs contact layer, 1.5-μm InP cladding layer, a pair of 40-nm AlGaInAs transition layers separating an InP layer from a separated confinement heterostructure layer, a pair of 75-nm AlGaInAs confinement layers, six quantum wells sandwiched between barrier layers, and a 200-nm n-InP contact layer, 60-nm InP etch stop layer, and 1-μm InGaAs sacrificial layer. The InP-SOA was printed onto the Si substrate after releasing the SOA coupon. The measurement indicated that more than 40 dB SMSR and 2.2 mW single-sided waveguide-coupled power were achieved at 70 mA.

To overcome the output power limitations of this Ⅲ–Ⅴ-on-Si DFB laser, the same group proposed TP-enabled DFB laser co-integration with an OPA. The amplifier featured a 780-μm-long straight waveguide section and two 180-μm alignment-tolerant Ⅲ–Ⅴ taper waveguides. The stand-alone DFB laser exhibited a 4-dBm waveguide-coupled output power and a 34-dB SMSR at 1540 nm. Following OPA co-integration, the waveguide output power reached 14 dBm, at the cost of a reduced SMSR (28 dB). This co-integration indicates the reliability and feasibility of transferring Ⅲ–Ⅴ devices onto SiPh platforms. Moreover, SOA coupons can be densely fabricated on InP substrates and printed onto target substrates with larger pitch, optimizing expensive InP wafer use^101^.

Narrow-linewidth lasers can be used in various applications, e.g., long-distance optical space communications and quantum measurement^105^. Thus, their integration is necessary to maintain SiPh applicability and marketability. In 2022, Soltanian et al.^87^ demonstrated a narrow-linewidth Ⅲ–Ⅴ-on-Si laser with a 110-nm wavelength tuning scope using TP. Prefabricated SOA coupons were printed on two Si-laser external cavities with photoluminescence peaks at ~1500 and 1550 nm. The combination of two independent extended laser cavities in a single-mode waveguide functioned as the laser output, yielding a widely tunable laser. Under 120-mA bias, the combined lasers exhibited a 110 nm wavelength tuning ability, from 1495 to 1605 nm.

In 2020, Loi et al.^102^ reported heterogeneous integration of an O-band Fabry-Perot InP laser into a SOI recess using TP technique. An SU8 polymer waveguide was edge-coupled to the laser and evanescently coupled to a tapered SOI waveguide. A calibrated metal layer was deposited between the laser and an SiO_2_ substrate to improve the SOI/laser vertical alignment accuracy; this layer was both a metal contact and a thermal via. The device light-current characteristics revealed a threshold current *I*_th_ ∼ 17 mA before and after TP to the SOI. *I*_th_ was increased to ∼ 23 mA following light coupling to the SU8 waveguide because of the reduced front-mirror reflectivity (from *R* ∼ 32% (in air) to *R* ∼ 15.5%). Efficient light coupling among a Si waveguide, SOI taper, and polymer waveguide may enable active waveguide-based PICs.

In 2023, Uzun et al.^103^ compared the performance of an O-band InAs/GaAs quantum-dot edge-emitting laser integrated onto different waveguide platforms (including 220-nm Si, 3-μm Si, and 300-nm SiN) using TP. For an elastomeric stamp retrieving laser coupons from the growth substrate and printing them onto the target substrate, the lateral and longitudinal misalignments were within 150 nm. The waveguide- and epitaxial-layer- thicknesses were varied to control the vertical alignment. The waveguide/laser coupling efficiencies were 7.5%, 12%, and 7% for the 220-nm Si, 3-μm Si, and 300-nm SiN waveguides, respectively. These values were affected by the position misalignment, the refractive index of the laser/waveguide gap, and laser/waveguide mode size mismatch. The TP yield exceeded 90% and the laser performance was uncompromised^103^.

VCSEL integration into a PIC enables high-throughput wafer-scale testing, a sub-mA lasing threshold, high slope efficiency, etc. In 2021, Goyvaerts et al.^104^ demonstrated TP-based integration of an 850-nm GaAs-based VCSEL coupled to a grating into a SiN PIC recess, yielding a vertical cavity surface integrated laser. The VCSEL comprised two DBRs and an intermediary MQW gain section. The bottom-emitting light from the VCSEL was coupled to the SiN waveguide through a diffraction grating. A waveguide-coupled power exceeding 100 μW and sub-mA lasing threshold were measured; the slight difference in waveguide-coupled power was due to misalignment during printing. The integrated laser exhibited strong wavelength tunability with an SMSR exceeding 45 dB.

### Other optical components

**Fig. 17:**
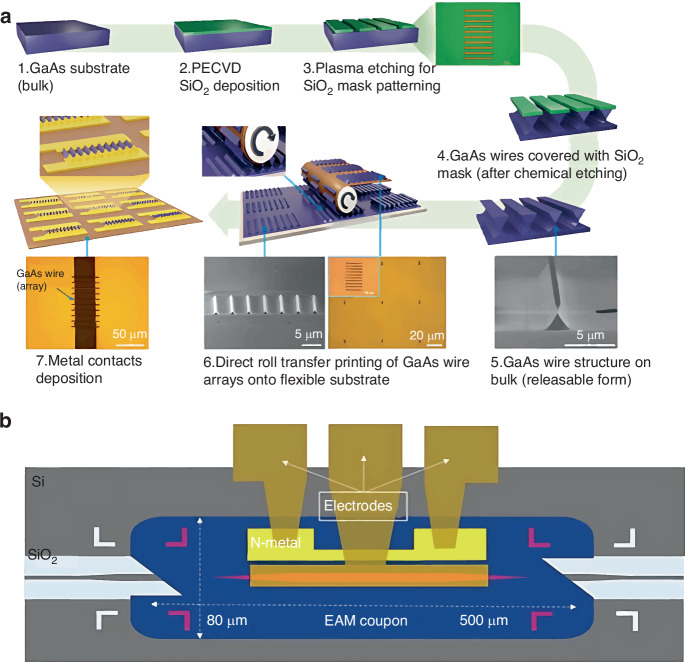
TP process of GaAs photodetectors and taper design of EAM devices. **a** Fabrication and TP process of GaAs photodetectors and related optical and SEM images^108^. Reproduced with permission. Copyright 2022, John Wiley and Sons. **b** EAM-device design schematic, part-fabricated EAM is printed onto the SOI waveguide and taper design aims for efficient light coupling between active region of EAM and SOI^116^. Reproduced with permission. Copyright 2024, AIP Publishing

Photodetectors are the most common opto-electronics components^106,107^. However, integrating these compound semiconductor-based micro/nanostructures onto flexible substrates is challenging. The transferred microscale GaAs photodetector arrays onto glass tubes using TP technology was reported in 2022^108^. Via a PI interlayer, a donor substrate with GaAs photodetector arrays closely contacted the target substrate to achieve TP without an elastomeric stamp (Fig. 17a). The strong adhesion state of the semi-cured PI layer ensured a high GaAs-photodetector yield (~ 95%). The transfer-printed GaAs photodetector arrays exhibited excellent performance under UV and near-infrared illumination, including high response (2.5 ms), and recovery times (8 ms), high responsivity (>10^4^ A W^−1^), detectivity (>10^14^ Jones), external quantum efficiency (>10^6^), and photoconductive gain (>10^4^) at 1 V. This non-stamp TP method using a spin-coated PI film onto a target substrate may enable microscale semiconductor-device integration into a flexible substrate. Further, fabrication costs are reduced because the donor substrate can be reused many times following device release.

In 2023, Muthuganesan et al.^109^ demonstrated TP of 21 × 57 μm^2^ InGaAs photodetectors onto 500-nm-thick SOI substrate using dual sacrificial layers, InGaAs and InAlAs. A PDMS stamp with posts of 20$\times$50 μm^2^ picked suspended devices from the donor substrate and printed them onto SOI substrate. Photodetector responsivity of 0.6 A W^−1^ with 48-nA dark current and 3-dB bandwidth of 17.5 GHz were observed, conforming to simulation, indicating that TP is promising for integration of compact high-bandwidth Ⅲ–Ⅴ devices onto PICs.

A transfer-printed InP-based thin-membrane LED can act as a single-spatial-mode broadband light source for sensing. In 2016, De Groote et al.^110^ proposed the first LED printed on and coupled with a PIC. High-contrast InP membranes were transferred from the InP substrate to SOI target substrate, and optically pumped LEDs were fabricated in the transferred material. Hence, 3-dB bandwidth of 130 nm was achieved, comparable to previous results.

An O-band InP-InGaAs photodiode was printed onto a SiN platform. The dark currents were 47.5 nA and 0.5 μA at −1- and −3-V bias voltages, respectively. The responsivity was increased to 0.9 A W^−1^ at 1310 nm with an applied bias voltage of −3 V^111^.

Electronic absorption modulators (EAMs) are essential for PICs, particularly for high-speed optical communications and sensing^112–115^. In 2024, Moynihan et al.^116^ transferred a high-speed EAM from an InP substrate to a 220-nm SOI (Fig. 17b). A tapered region in an InP ridge ensured evanescent light coupling between the Ⅲ–Ⅴ waveguide and SOI for efficient transmission. The printed EAM had a 30-dB maximum extinction ratio (ER), 40-GHz electronic bandwidth, and 6.5-dB insertion ratio at 1550 nm.

Fast modulators are essential for advancing integrated photonic systems to keep up with the demanding operation speed and data rates^117^. The first high-speed thin film lithium niobate (TFLN) modulator heterogeneously integrated on SiN waveguide via the TP method was demonstrated in 2023^118^. Adiabatic tapers on both ends of TFLN waveguide were designed to reduce the index mismatch between SiN and hybrid waveguides, realizing a 39 dB ER, 3.3 dB insertion loss and 70 Gb s^−1^ data generation capability, indicating TP-based approach a promising pathway for high performance and miniaturized modulators. A transfer-printed TFLN-on-Si ring modulator for the dense integration of TFLN modulator with compact Si circuit was also presented for the first time in 2024^119^, which could support 45 Gbit s^−1^ signals with −37 dB of ER. Photonic research group at Ghent University^120^ also printed 1 cm-long TFLN onto SiN waveguide and integrated it into a push-pull Mach-Zehnder modulator with at least 35 GHz modulation speed.

Heterogeneous integration of TFLN onto SiN platform also enables narrowband high-speed filters. Leveraging the low-loss characteristics of SiN waveguides and high-speed electro-optic phase shifting of TFLN, Su et al.^121,122^ proved the TP-based large-area heterogeneously integrated tunable micro-ring filter with less than 3 ns response time and 1.2 GHz of optical bandwidth. Compared with previously reported results^123,124^, their filter achieves higher loaded Q-factor, improved ER values, and larger coupon sizes (up to 230 × 2000 μm^2^), overcoming the area limitations of the traditional TP process.

As key elements in the datacenter networks, optical switches can be achieved with TFLN-on-SiN waveguide platform via the Pockels effects of TFLN with negligible loss. In 2022, Zhang et al.^125^ realized an array of C-band Ⅲ–Ⅴ amplifiers co-integrated with Si MZI switch. High-speed switching was verified by routing an external 12 Gb s^−1^ data stream through the array. A 2 × 2 cascaded electro-optic switch using the transfer-printed TFLN-on-SiN platform was developed in 2025^126^ with <3 ns of response time, <-45 dB of crosstalk and >100 nm of 3-dB bandwidth.

TP enables integration of III–V optical-electronic devices onto PICs for broader applicability. III–V devices can be fabricated on their growth substrate, InP or GaAs, and Si/SiN PICs can be realized in open-access CMOS foundries, enabling TP-based heterogeneous device integration. This section lists several sample Ⅲ–Ⅴ devices integrated onto PICs, including SOAs, lasers, photodetectors, LEDs, photodiodes, EAMs, phase modulators, tunable filters and optical switches (Table 3). The excellent device performance after TP validates this approach for deterministic microscale-device assembly.

**Table 3:** Summary of integrated III–V devices discussed herein

Time	Device	Integration platform	Crucial parameter	TP tool	Ref.
2019	C-band SOA	Si	Gain = 17 dB, on-chip peak output power = 10 dBm@170 mA	X-Celeprint μTP-100	^91^
2020	C-band SOA	Si	Gain = 23 dB, on-chip saturation power = 9.2 mW@140 mA (high Γ) Gain = 17 dB, on-chip saturation power = 15 mW@160 mA (low Γ)	X-Celeprint μTP-100	^92^
2023	SOA@ 1573 nm	Si	Gain = 9.4 dB, output saturation power = 15.4 dBm@ 114 mA (left), 140 mA (right)	/	^93^
2020	SOA@ 1550 nm	SiN	Gain = 14 dB, output saturation power = 8 mW@120 mA	X-Celeprint μTP-100	^94^
2012	Fabry-Perot laser@ 824 nm	Si	Modulation bandwidth >3 GHz, total optical power >60 mW, operating temperature >100 °C	PDMS stamp with well-defined posts	^97^
2012	Membrane reflector VCSEL	Si	Output power ~10 μW, power efficiency <0.1%	PDMS stamp	^98^
2018	DFB laser@ 1550 nm	Si	SMSR > 40 dB, waveguide-coupled output power = 2.2 mW@70 mA	X-Celeprint μTP-100	^100^
2023	Co-integration of DFB laser and OPA	Si	SMSR > 28 dB, waveguide-coupled output power = 14 dBm@270 mA	PDMS stamp with a single post with 40 µm × 1200 µm	^101^
2022	Narrow-linewidth laser	Si	Wavelength tuning scope >100 nm	X-Celeprint μTP-100	^87^
2023	O-band QD-laser	SOI/SiN	Waveguide-coupled power = 1 mW@85 mA (220 nm SOI), 0.95 mW@85 mA (300 nm SiN), 1.7 mW@85 mA (3 μm SOI)	Single PDMS stamp	^102^
2021	VCSEL@ 850 nm	SiN	SMSR >45 dB, waveguide-coupled output power >100 μW	X-Celeprint μTP-100	^104^
2022	GaAs photo-detector	Glass	Response time = 2.5 ms, recovery time = 8 ms, responsivity >10^4^ A W^−1^, detectivity >10^14^ Jones, external quantum efficiency >10^6^, photoconductive gain >10^4^@1 V	PI interlayer	^108^
2023	InGaAs photo-detector	SOI	Responsivity = 0.6 A W^−1^@48 nA dark current, 3-dB bandwidth = 17.5 GHz	PDMS stamp with post size of 20 × 50 µm^2^	^109^
2016	LED	SOI	3-dB bandwidth = 130 nm	Patterned PDMS stamp with posts	^110^
2024	O-band InP-InGaAs photodiode	SiN	Responsivity = 0.9 A W^−1^@ −3 V, 1310 nm	/	^111^
2024	EAM@ 1550 nm	SOI	Electrical bandwidth = 40 GHz, ER = 30 dB@−6–0 V	80 × 500-μm^2^ PDMS stamp	^116^
2023	TFLN modulator	SiN	Half voltage = 14.8 V, insertion loss = 3.3 dB, ER = 39 dB, 3-dB bandwidth >50 GHz	/	^118^
2024	TFLN ring modulator	Si	Insertion loss = −1.5 dB, ER = −37 dB, electro-optical bandwidth = 16 GHz, modulation rate = 45 Gbit s^−1^	/	^119^
2025	TFLN-on-MZI modulator	SiN	Half voltage = 3.2 V, propagation loss = 0.9 dB cm^−1^, transition loss = 1.8 dB facet^−1^	PDMS stamp	^120^
2025	TFLN micro-ring optical filter	SiN	3-dB bandwidth = 1.2 GHz, tuning efficiency = 2 pm V^−1^, response time <3 ns, static ER > 20 dB, Q-factor = 10^5^	A PDMS stamp with a racetrack-shaped post	^121,122^
2022	MZI switch	Si	Optical gain = 10 dB, 3-dB bandwidth >30 nm @SOA, improvement of optical cross-talk suppression = 56 dB	X-Celeprint μTP-100	^125^
2025	Cascaded TFLN optical switch	SiN	3-dB bandwidth >100 nm, crosstalk <−45 dB, response time <3 ns	/	^126^

## Conclusions and perspectives

Advances in TP techniques and their implementation in PICs are summarized. TP is a highly versatile and potent method for the precise and deterministic assembly of a wide range of microscale devices. A suitable stamp and modification method are essential for nondestructive transfer. The feasibility and reliability of TP-based heterogeneous integration of III–V devices onto PICs were demonstrated. Combining the rate-dependent adhesion effect of the stamp with shear-enhanced TP provides a reliable method of integrating non-native Ⅲ–Ⅴ devices onto PICs for broader applicability. TP-based integration of SOAs, lasers, and other optical-electronic components was discussed, indicating the feasibility and reliability of TP. However, most of the summarized TP techniques were realized in the laboratory; therefore, great efforts need to be committed to bring these techniques towards maturity. Further, comprehensive characterization of TP-based devices is required.

Although TP has low-cost and high-fidelity properties, issues remain. First, a commercial state-of-the-art TP tool can achieve $\pm$1.5- and $\pm$0.5-μm alignment accuracy for device arrays and individual device coupon^127^, respectively; thus, the overall TP yield and alignment accuracy require improvement. Regarding PICs, use of adiabatically tapered structure to handle misalignment during TP has been proposed. However, the alignment accuracy must be enhanced to ensure efficient light coupling from and to Ⅲ–Ⅴ devices. Additionally, nano-scale device transfer onto a target substrate is challenging due to the microscale stamp design. Finally, tradeoffs must be made between cost, fabrication difficulty, throughput, and operation time; thus, a widely compatible stamp is required for industrialization.

TP technique is still at an early stage, necessitating further refinement and extending to a broader applicable range in the future. The realizations of on-chip broadband spectrometer with picometer scale resolution^128,129^, on-chip signal processing^130^ and photonic quantum computing are promising proof-of-concepts that highlight the potential of TP technology in other application domains, such as reconfigurable photonic systems, flexible electronics^131,132^ and biocompatible electronics^133–135^. The integration of pre-fabricated nano-scale optical devices (e.g., nano-wires, nano-grating and optical vortexes)^136^ onto PIC platform via TP is also beneficial for the establishment of powerful, complex and cost-efficient nanophotonic systems. This review suggests that TP research will actively continue, promising further innovation and progress.

## Data Availability

My article does not present original research.
